# *Streptococcus uberis* strains isolated from the bovine mammary gland evade immune recognition by mammary epithelial cells, but not of macrophages

**DOI:** 10.1186/s13567-015-0287-8

**Published:** 2016-01-07

**Authors:** Juliane Günther, Anna Czabanska, Isabel Bauer, James A. Leigh, Otto Holst, Hans-Martin Seyfert

**Affiliations:** Institute for Genome Biology, Leibniz Institute for Farm Animal Biology (FBN), Wilhelm-Stahl-Allee 2, 18196 Dummerstorf, Germany; Division of Structural Biochemistry, Research Center Borstel, Leibniz-Center for Medicine and Biosciences, Parkallee 1-40, 23845 Borstel, Germany; Department Animal Health and Welfare, School of Veterinary Medicine and Science, University of Nottingham, Sutton Bonington, Leicestershire, LE12 5RD UK

## Abstract

*Streptococcus uberis* is frequently isolated from the mammary gland of dairy cattle. Infection with some strains can induce mild subclinical inflammation whilst others induce severe inflammation and clinical mastitis. We compared here the inflammatory response of primary cultures of bovine mammary epithelial cells (pbMEC) towards *S. uberis* strains collected from clinical or subclinical cases (seven strains each) of mastitis with the strong response elicited by *Escherichia coli*. Neither heat inactivated nor live *S. uberis* induced the expression of 10 key immune genes (including *TNF*, *IL1B*, *IL6*). The widely used virulent strain 0140J and the avirulent strain, EF20 elicited similar responses; as did mutants defective in capsule (*hasA*) or biofilm formation (*sub0538* and *sub0539*)*. Streptococcus uberis* failed to activate NF-κB in pbMEC or TLR2 in HEK293 cells, indicating that *S. uberis* particles did not induce any TLR-signaling in MEC. However, preparations of lipoteichoic acid (LTA) from two strains strongly induced immune gene expression and activated NF-κB in pbMEC, without the involvement of TLR2. The immune-stimulatory LTA must be arranged in the intact *S. uberis* such that it is unrecognizable by the relevant pathogen receptors of the MEC. The absence of immune recognition is specific for MEC, since the same *S. uberis* preparations strongly induced immune gene expression and NF-κB activity in the murine macrophage model cell RAW264.7. Hence, the sluggish immune response of MEC and not of professional immune cells to this pathogen may aid establishment of the often encountered belated and subclinical phenotype of *S. uberis* mastitis.

**Electronic supplementary material:**

The online version of this article (doi:10.1186/s13567-015-0287-8) contains supplementary material, which is available to authorized users.

## Introduction

The Gram-positive bacterium *Streptococcus uberis* is among the four most prevalent species of mastitis causing pathogens [1, 2]. Infection with this bacterium can occur with very few if any clinical signs, but can also result in severe inflammation of the udder culminating in clinical mastitis [3]. It is not entirely clear if the heterogeneous physiology of *S. uberis* mastitis is caused by genetic diversity of the different strains infecting the udders. There is an extraordinary diversity of *S. uberis* strains [4, 5] and clear examples of strains that reproducibly induce clinical or subclinical infections have been reported [5–7]. There are reports that occasionally a predominant strain infected several cows within a herd [8] but it was also found that genetically distinct *S. uberis* strains infected different individuals within a herd and distinct strains caused re-infection after a previous successful cure of a first *S. uberis* infection [9, 10].

Multilocus sequence typing studies suggested that mastitis causing *S. uberis* strains (clinical and subclinical) may be genetically different from avirulent strains [11]. However, no clear gene-loss or -gain correlation with the virulent or avirulent phenotype of the strains emerged in a very recent comparison of the whole genome sequences from thirteen different *S. uberis* strains [5]. This suggests that the particular outcome of an udder infection is largely determined by the host-pathogen interaction rather than by the particular genotype of the pathogen.

*Streptococcus uberis* vs. cow interactions have been studied in several udder infection experiments. *S. uberis* generally elicited a belated onset of inflammation, compared to infections with *E. coli* or other Gram-negative pathogens [12, 13]. Comparing global transcriptome profiling from *S. uberis* vs. *E.* *coli* infection trials reveals a remarkable failure of *S. uberis* to induce expression of pro-inflammatory cytokine and chemokine-encoding genes in the udder [14–19]. It rather appeared that *S. uberis* infection up-regulated IL10 and IL6 governed pathways, which are both known to eventually counteract strong inflammation [15]. Moreover, these studies recapitulated the physiological variability in the outcome of *S. uberis* udder infections. While the widely used strain 0140J—known as the almost prototypical strain for eliciting clinical mastitis [6, 20]—caused clinical mastitis in one of these infections trials [15], it elicited subclinical mastitis in the second trial [16].

Mammary epithelial cells (MEC) of the lactating udder parenchyma are the dominant cell type coming into contact with invading pathogens early on after infection. Quantitative morphometry revealed that MEC comprise more than 70% of the udder cells [21, 22]. MEC are the dominant sentinels of the lactating parenchyma and competent to mount the first cytokine alert [17, 23–25]. They express not only the relevant Toll-like-receptors (TLRs) for perceiving pathogens but also β-defensin-encoding genes to counteract alveolar colonization of the pathogens [26–28]. It was reported that challenging these cells with only some strains of *S.* *uberis*, but not with others, would activate cytokine genes expression in these cells [14, 29].

Epithelial cells are known to perceive pathogens through pathogen recognition receptors (PRR) including the family of TLRs [30]. Thirteen mammalian TLRs are known. Binding of their ligands (collectively known as Pathogen Associated Molecular Patterns; PAMPs) activates their downstream signaling. This ultimately leads to the activation of the NF-κB factor complex, mediated through a multifactorial cascade [31]. These transcription factors regulate the expression of a wealth of immune genes [32–34]. TLR2 is known to be essential for mounting an efficient immune defence against Gram-positive bacteria [35–37]. Lipoproteins from these pathogens belong to its natural ligands [38, 39]. However, it was reported that Gram-positive Group B streptococci (e.g. *S. agalactiae*) did not activate TLR2 [40]. Moreover, mitomycin-C inactivated *S. uberis* preparations did not activate the bovine TLR2 receptor in HEK293 cells [41]. Failure to activating a TLR mediated immune response would readily explain the slow and weak immune response caused by a *S. uberis* challenge.

We wanted to know if the divergent physiology of *S. uberis* mastitis might in tendency be related to *S. uberis* strain specific differences in the interaction with the MEC. Therefore, we wanted to establish a broader survey of the capacity of diverse *S. uberis* isolates from clinical and subclinical cases of mastitis to stimulate immune functions of MEC. We included previously used model strains either known to causing mastitis or as being avirulent for direct comparison of the results with current knowledge. We were also curious about the possible divergent immune stimulatory properties of cell wall components isolated from different strains to see if they would possibly cause a strain dependent difference in eliciting an immune response.

Our parameters for the induction of immune functions consisted of the pathogen challenge -related modulation of the expression of a panel of cytokine- and chemokine-encoding genes (such as TNF, IL1A, IL6, CXCL8) but also those encoding effectors of immune defence (β-defensin LAP, NOS2A) or membrane protecting factors (SAA). Their relevance as markers for induced immune functions in MEC has previously been established [17]. Additionally, we monitored the activation of the NF-κB transcription factor complex. We show that—under our experimental conditions—all the *S. uberis* strains failed to significantly induce immune functions in the MEC, but not in macrophage model cells.

## Materials and methods

### Mastitis pathogen strains

*Streptococcus uberis* strains used in this study are 233 (obtained from the AgResearch New Zealand [14]), 0140J (ATCC^®^ Number BAA-854, isolated from clinical mastitis, 1972 in the UK), EF20 (avirulent strain isolated from clinical mastitis, 1970 in the UK [6], Δ*has*A (noncapsular hyaluronate synthase A mutant derivative of strain 0140J; [42]); 0140J::IS*S1* P’ (mutant of 0140J, inactivation of the promoter addressing the glycosyltransferase genes sub0538 and sub0539 which significantly reduced the ability to form biofilm [43]); T1–18 and T2–58 (isolated from cases of mastitis in the UK, provided by Dr Michael Fontaine, Moredun Research Institute, UK). The *S. uberis* strains C6344, C5072, S6261, C9359, Ab71 (all isolated 2002 in the UK), 4428 (1999, UK), 5291 (2000, UK) were isolated from clinical cases while C8329, C5388, C7131, S7010 (all isolated 2002 in the UK), B190, B362 (2000, UK), 6736 (1999, UK) were isolated from subclinical cases of mastitis. *S. agalactiae* 0250 and *S. dysgalactiae* 2023 were isolated from cases of bovine mastitis in the UK and are included in the National Institute for Research in Dairying culture collection (currently hosted by Prof. James Leigh at the University of Nottingham). *E. coli* strain 1303 is a well characterized mastitis model strain isolated from udder secretions of a cow with clinical mastitis [19].

### Bacterial growth and preparation of pathogen particles

Details regarding culturing *E. coli*_1303_ and its use to challenge the pbMEC were exactly as described [17]. *S. uberis*, *S. agalactiae* and *S. dysgalactiae* were grown in Todd Hewitt Broth (THB, Carl Roth GmbH) at 37 °C without agitation to the logarithmic phase of culture growth (0.5, OD_600_ nm). Plating of dilution series was used to calibrate cell counts from the OD readings. Efficacy of killing the bacteria through heat treatment (60 min, 80 °C) was verified by control plating. Heat treated cells were collected by centrifugation, washed twice with RPMI 1640 medium (Biochrom), and re-suspended therein. Aliquots were stored frozen at −20 °C. We applied in challenge experiments similar protein concentrations of the heat killed bacteria from the different strains in order to standardize the conditions. Protein contents of the bacterial preparations had been determined with the Lowry procedure [44]. Based on three independent growth experiments, we found from exponentially multiplying cultures (OD_600nm_, 0.5) as protein content ~16.8 ± 4.1 and ~5.8 ± 0.8 µg/10^7^ bacteria for of *E. coli*_*1303*_ and *S. uberis* strain 0140J, respectively. The other *S. uberis* strains had protein contents similar that determined for 0140J.

### LTA preparation

LTAs were isolated as described by Morath et al. [45] with few modifications as detailed [46]. Treatment of lyophilized native LTA with 1% H_2_O_2_ in phosphate-buffered saline (PBS) inactivated contaminating lipoproteins [47]. Our NRM spectroscopy analysis proved that the LTA core structure was not altered by that treatment. LTA preparations were endotoxin free as judged by their failure to activate the bovine TLR4 in the HEK293 cells. The latter assays were conducted as previously described [27].

### Cell culture procedure

Tissue cultures of pbMEC were established as described [27]. Their cultivation and pathogen stimulation on collagen IV coated tissue culture plates in RPMI 1640 (Biochrom) supplemented with insulin, prolactin, dexamethasone and 10% FCS (PAN-Biotech) was as detailed by Günther et al. [17]. Briefly, frozen aliquots of pbMEC were seeded at high cell density into 9 cm dishes, purified through selective trypsinization and reseeded for experiments into six well plates, again at high cell density. After settling overnight duplicate wells were challenged at various times (t0, t21, t23 h) through the addition of 30 µg/mL of protein from heat killed bacteria. Duplicate unstimulated control cultures were kept in parallel. All cultures were collected at t24 h for RNA extraction.

RAW264.7 cells (from ATCC) were cultivated in DMEM (Biochrom) supplemented with 2 mM l-glutamine and 10% FCS. Stimulation experiments were similarly performed using 80% confluent cell cultures.

All tissues and cells were retrieved from healthy first lactating Holstein–Friesian heifers having been slaughtered in our local abattoir, complying with all pertinent ethical and legal requirements. The abattoir is a EU licensed (ES1635) core facility of the research affiliation and serves to routinely supply samples to different laboratories. Special ethical approval was unnecessary since the cows had been culled in the normal culling regime without conducting any animal experimentation.

### RNA extraction and mRNA quantification

RNA was extracted with TRIZOL-reagent (Invitrogen). Preparation of the cDNA (Superscript II, Invitrogen) and real time quantification of the mRNA concentrations with the Fast-Start Sybr Green I kit and the LightCycler II instrument (Roche) were done essentially as described [26], however using per assay a cDNA input derived from 75 ng of total RNA. Titration of relative copy numbers against external standards and normalization against the not regulated reference gene chloride intracellular channel 1 (CLIC1) were done as detailed in [48]. Sequences of oligo nucleotide primers are listed in Additional file 1.

### Determination of NF-κB activation

NF-κB activity was assessed with a reporter gene expressing the Renilla-luciferase under the control of the NF-κB activated ELAM promoter (Invivogen; [27]). This reference describes also the vector expressing the bovine TLR2 receptor. These constructs were transfected into pbMEC and HEK293 cells with Lipofectamine 2000 (Invitrogen) essentially as previously described in detail [49].

Macrophages are notorious for being difficult to transfect due to the natural response of phagocytes against foreign materials. Therefore RAW264.7 cells were transfected using the Neon^®^ Transfection System (Life Technologies) following the manufacturer’s instructions for this specific cell type. Briefly 5 µg of the ELAM NF-κB reporter plasmid were used to transfect 10^6^ cells with one pulse of 1580 V for 20 ms. Subsequently the cells were seeded into wells of 24-well plates. The cells were allowed to recover overnight prior to stimulation. After challenging with the respective stimulus for the time as indicated, the cells were lysed and luciferase activity was assayed using the dual luciferase assay reporter system (Promega) as detailed [27]. The enzyme activity was calibrated against the protein content of the lysate.

### Stimulation with live pathogens

Pathogens were grown to the logarithmic phase of culture growth (0.5, OD_600nm_) in the respective growth medium. Subsequently, the bacteria were washed twice with RPMI 1640 medium and resuspended therein. They were co-cultured with pbMEC in RPMI 1640 medium (without antibiotics) for 1 h with 10^7^ CFU/mL of the respective pathogen. Subsequently pathogens were killed by adding 100 µg/mL gentamicin. For mRNA quantification the pbMEC were either instantly harvested (1 h time point) or cultured for another 2 or 23 h in pbMEC growth medium (3 and 24 h time point, respectively). For luciferase measurement of NF-κB activation lysates were prepared 23 h after bacterial killing with gentamicin.

### Statistical analysis

The data were analysed with GraphPad Prism Version 5 (GraphPad Software, Inc., La Jolla, CA, USA). Differences were evaluated through an analysis of variance (ANOVA) including Bonferroni’s correction for pairwise multiple comparisons.

## Results

### *Streptococcus uberis* strains failed to activate immune gene expression in pbMEC

**Figure 1:**
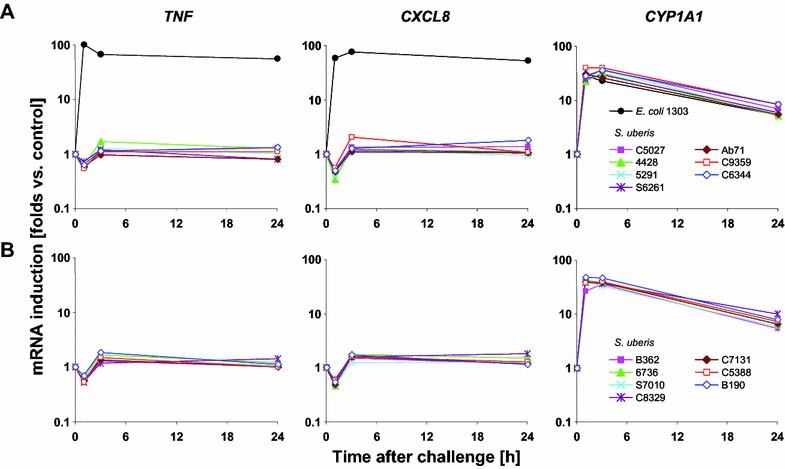
***Streptococcus uberis*** **strains from clinical or subclinical cases of mastitis did not activate immune functions of pbMEC.** Induction of gene expression (ordinate) of *TNF*, *CXCL8* and *CYP1A1* after challenging for 0, 1, 3 and 24 h (abscissa) with 30 µg/mL of 14 different heat-killed *S. uberis* strains isolated from clinical (**A**) or subclinical (**B**) cases of mastitis compared against *E.* *coli*_1303_. Values are RT-qPCR measurements of the respective mRNA species, normalized against CLIC1 and expressed as multiple of the value measured from the unstimulated control. Values are from a single experiment, assayed in duplicate. Only *E. coli*_1303_ elicited a significant induction (compared to control, *p* < 0.05) of *TNF* and *CXCL8*. All preparations strongly induced the expression of *CYP1A1*.

Dose finding studies had confirmed in pilot experiments that preparations of the heat-killed particles from the non-encapsulated strain 233 did not significantly induce cytokine gene expression in pbMEC [14], even if applied at high concentrations (up to 10^8^ particles/mL; data not shown). The pathogen concentration in the milk of *S. uberis* infected udders is known to reach ~10^7^ CFU/mL [5, 13]. We then surveyed the immune stimulatory properties of a broader collection of strains isolated from either clinical or subclinical cases of mastitis to eventually find indications for strain specific differences. pbMEC cultures were stimulated for up to 24 h with 30 µg/mL of protein from heat-killed preparations of seven different strains each isolated from clinical or subclinical cases of mastitis. Thus, approximately 100 bacterial particles were applied per MEC host cell (MOI ~100). A similarly preparation of *E. coli*_1303_ was included as a positive control. Contrary to *E. coli*, all *S. uberis* strains failed to significantly activate gene expression of seven different immune genes (*TNF*, *CXCL8* (Figure 1), *IL1A*, *IL6*, *CCL5*, *SAA3*, β-defensin *LAP*; data not shown). No difference was observed between isolates obtained from clinical or subclinical infection. However, expression of *CYP1A1* was strongly induced to a similar extent by all bacterial preparations (Figure 1), validating that the cells had perceived presence of disturbing compounds in their environment. Expression of this general detoxification enzyme [50] is induced by a wide variety of xenobiotic stress and largely regulated by the aryl hydrocarbon receptor (AhR) and its nuclear translocator (ARNT; see [51, 52] for reviews).

 We repeated the survey using another two different mastitis isolates, strain 233 and the widely used strain 0140J. Again, no significant induction of the candidate immune genes was recorded (Additional file 2) and these data validate that the absence of immune stimulatory properties of the model strains 0140J and 233 on MEC are typical for mastitis causing *S. uberis* pathogens.

**Figure 2:**
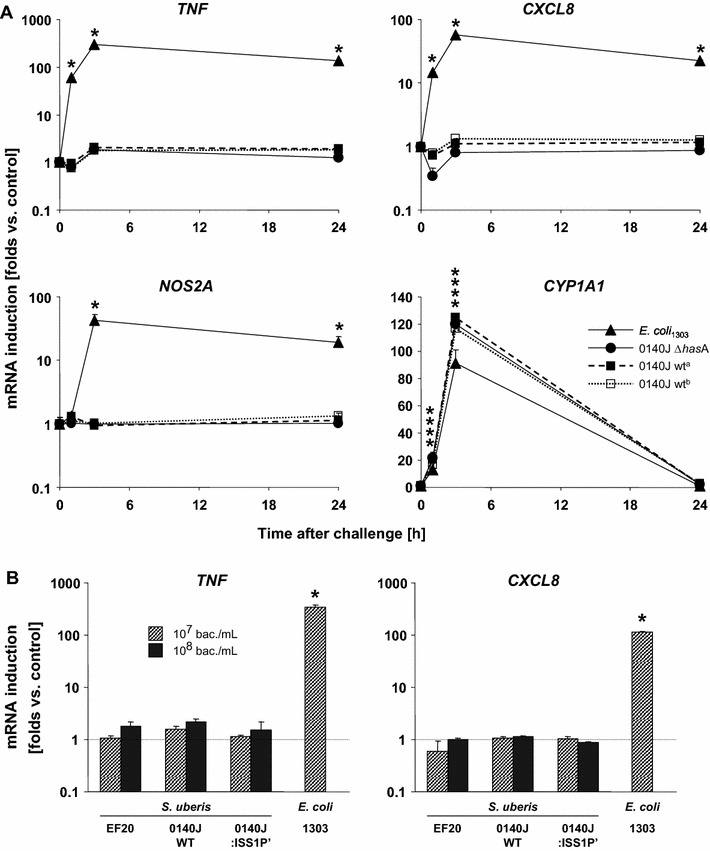
**Neither** ***S. uberis*** **mutants with defects in the formation of a hyaluronic acid capsule or in glycolipid biosynthesis nor an avirulent strain activated immune functions of pbMEC.** **A** Induction of gene expression (ordinate) of *TNF*, *CXCL8* and *NOS2A* after challenging with 30 µg/mL of heat-killed 0140J *has*A mutant (Δ*has*A) compared against two different preparations of wild-type 0140J (0140J wt^a/b^) and *E.* *coli*_1303_ for the time as indicated (abscissa). All preparations strongly induced the expression of *CYP1A1*. Values are from a single experiment representative for three, each assayed in duplicate. **B** Same experimental setting as before, but the challenge time was 3 h only. EF20, avirulent strain; 0140J::IS*S1* P’, mutant defective for glycolipid biosynthesis. The experiment was assayed in triplicate. Asterisks indicate significance of the different mean values from the control (*p* < 0.05).

As the presence of the capsule is positively correlated with isolates from clinical disease [53], we next examined if the capsule might be responsible for the poor immune stimulatory properties of *S. uberis* on MEC. The Δ*has*A mutant of strain 0140J cannot form capsule [42]. We found that it was as ineffective in activating an immune response in pbMEC as the wild type strain (Figure 2A). Similarly ineffective was a mutant of *S. uberis* strain 0140J with a fivefold reduced capacity to express a key gene involved in glycolipid formation and proven reduced capacity for biofilm formation (Figure 2B).

We have recently observed that addition of 10% FCS greatly quenched the reactivity of pbMEC against *Staphylococcus aureus*, another representative of a Gram-positive pathogen [48]. Hence, we examined the effect of FCS supplementation on the pbMEC response against *S. uberis*. Therefore the pbMEC were grown in normal growth medium. They were then washed twice with PBS and growth medium devoid of FCS was added. The cells were challenged in this medium for three h with *S. uberis*_233_, *E.coli*_1303_ and similar preparations of *S. aureus*_1027_. *E. coli* and *S. aureus* quite strongly induced the expression of most of our candidate immune genes. However, the response against *S. uberis* was to that recorded in normal growth medium (Additional file 3).

### *Streptococcus uberis* did not quench the reaction of pbMEC against *E. coli*

It has been reported that components of the *S. uberis* capsule may impair neutrophil functions [53]. We therefore examined, if pre-incubating pbMEC with *S. uberis* might impair the immune reactivity of the host cells. Cultures were pre-incubated for 1 h with 30 µg/mL of heat-killed particles from either of two *S. uberis* strains and subsequently challenged with a mild dose (3 µg/mL) of heat-killed *E. coli* particles. Scoring the mRNA concentrations of our panel of immune genes revealed no indication that pre-incubation reduced the immune response to *E. coli*; the expression of all these genes subsequent to the *E. coli* challenge was almost identical in *S. uberis* pre-incubated cultures and their respective controls (Additional file 4).

### Live *S. uberis* elicited only a slightly enforced immune reaction

Any method to inactivate pathogens alters the surface composition of the particles. Hence, we analyzed if co-culturing the pbMEC with live pathogens would hasten and inforce the immune reaction of the pbMEC. We therefore co-cultured these cells for 1 h with 10^7^ CFU/mL of *S. uberis* strain 0140J. Then the bacteria were killed through the addition of 100 µg/mL of gentamicin and the cultures were subsequently sampled at various times. Induction of immune gene expression was again found to be weaker by an order of magnitude than elicited by a similar challenge with *E. coli* (Table 1). However, live *S. uberis* pathogens induced the expression several genes (*TNF*, *IL6*, *CXCL8*) to a slightly larger extent than the heat-killed pathogens.

**Table 1:** **Extent and kinetics of modulated mRNA concentrations after stimulating pbMEC with live** ***E. coli***_**1303**_ **or** ***S. uberis*** **strain 0140J.**

Gene	Pathogen	Time		
		1 h	3 h	24 h
*TNF*	*E. coli* 1303	5 ± *0*	**88** ± *1*	34 ± *10*
	*S. uberis* 0140J	2 ± *1*	11 ± *3*	2 ± *1*
*IL6*	*E. coli* 1303	3 ± *1*	**34** ± *0*	7 ± *2*
	*S. uberis* 0140J	1 ± *0*	3 ± *0*	1 ± *0*
*CXCL8*	*E. coli* 1303	6 ± *0*	**213** ± *9*	**36** ± *5*
	*S. uberis* 0140J	1 ± *0*	**17** ± *10*	5 ± *2*
*CCL5*	*E. coli* 1303	1 ± *0*	14 ± *6*	**124** ± *85*
	*S. uberis* 0140J	1 ± *0*	1 ± *0*	1 ± *1*
*NOS2A*	*E. coli* 1303	4 ± *1*	**137** ± *11*	17 ± *6*
	*S. uberis* 0140J	1 ± *1*	7 ± *1*	1 ± *0*
*LAP*	*E. coli* 1303	1 ± *0*	5 ± *0*	**54** ± *8*
	*S. uberis* 0140J	1 ± *0*	1 ± *0*	2 ± *0*
*SAA3*	*E. coli* 1303	1 ± *0*	13 ± *1*	**72** ± *35*
	*S. uberis* 0140J	1 ± *0*	2 ± *0*	2 ± *1*
*CYP1A1*	*E. coli* 1303	77 ± *12*	**445** ± *61*	3 ± *1*
	*S. uberis* 0140J	79 ± *39*	**590** ± *122*	5 ± *1*

pbMEC were co-cultured for 1 h with 10^7^ CFU of the respective pathogen. Subsequently, pathogens were killed by adding 100 µg/mL gentamicin. mRNA was harvested either instantly (1 h time point) or after culturing for another 2 or 23 h (3 and 24 h time point, respectively). Values are means (±SEM) of fold change relative to the unstimulated control from two biological replica experiments, each assayed in duplicate. Numbers given in bold font indicate significant regulation

### *Streptococcus uberis* failed to elicit TLR2 signaling and NF-κB activation in MEC

**Figure 3:**
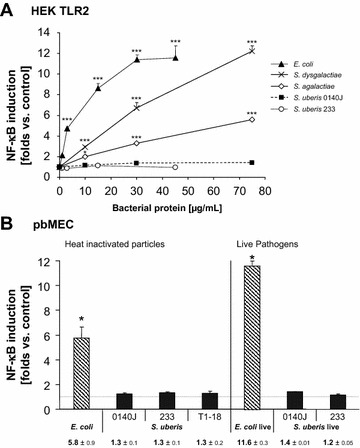
**Capacity of streptococcal pathogens to activate TLR2 and NF-κB. A** *S. uberis*, but not other streptococcal species failed to activate NF-κB in the HEK293 reconstitution system of TLR2-signaling. HEK293 cells were transfected with constructs expressing the bovine TLR2 receptor (200 ng) and the Renilla luciferase expressing reporter gene being driven by NF-κB through its ELAM promoter. Subsequently, the cells were challenged for 24 h with different dose (abscissa) of heat-killed streptococcal strains or *E. coli*_1303_. The luciferase activity was measured from cell lysates and normalized against their protein concentration. Values are expressed as fold increase above the level of the unstimulated control (ordinate). Each transfection was run in duplicate and assayed from triplicate challenges. (*p < 0.05; ***p < 0.001, regarding the difference to the unstimulated control). **B** *S. uberis* bacteria, dead or alive failed to activate NF-κB in pbMEC. pbMEC were transfected with the ELAM driven reporter gene construct (100 ng) and either stimulated with 30 µg/mL of the heat-killed bacteria (as indicated; left hand panel) or incubated for 1 h with 10^7^ live bacteria/mL. Bacteria were subsequently killed with 100 µg/mL gentamicin and the cultures incubated for another 23 h. Thereafter, the Renilla activity was measured from cell lysates and expressed as multiple of the respective unstimulated control (ordinate; tabulated values below the graph, mean ± SEM, *n* = 2 independent experiments, each assayed in triplicate). Only the NF-κB inductions in response to the *E. coli* challenges were statistically significant (*p* < 0.001).

We examined if a lack of TLR activation might be the underlying cause for the absence of any immune gene activation in MEC. On the one hand we inquired about the *S. uberis* mediated activation of the TLR2 receptor, since there is compelling evidence that this particular TLR is crucially involved in counteracting infection by Gram-positive pathogens [35, 38] and a previous report had suggested that TLR2 might not be activated by *S. uberis* [41]. HEK293 cells were co-transfected with our construct expressing the bovine TLR2 factor and the NF-κB driven luciferase reporter construct. Ligand mediated NF-κB activation would indicate TLR2 activation. None of the two *S. uberis* strains activated NF-κB in the HEK293 cells, even at very high concentrations of particles added (75 µg/mL equaling a MOI of approximately 300; Figure 3A). However, heat-killed particles of other streptococcal species (*S. dysgalactiae* and *S. agalactiae*) induced TLR2 to a similar extent as challenging with *E. coli*, which had been included as a positive control.

Pathogen-induced signaling from all TLR receptors is known to ultimately culminate in the functional activation of the NF-κB factor complex. Hence, we analyzed if *S. uberis* would at all activate NF-κB in pbMEC. In one set of experiments, we transfected the pbMEC cells with the NF-κB reporter construct and subsequently challenged the cells with heat-killed particles from three different stains. We found in all cases a 30% increase of the active NF-κB factors. However, the stimulations were statistically insignificant (Figure 3B, left hand panel). We repeated the experiment with live pathogens. The transfected cells were co-cultured with live pathogens (MOI, 30) for 1 h. Subsequently, the pathogens were killed (but kept in the culture fluid) by the addition of 100 µg/mL of gentamicin and the NF-κB activity was assayed 24 h later. The result was similar. Challenge with live *S. uberis* pathogens (strains 0140J and 233) did not significantly increase the level of active NF-κB (Figure 3B, right hand panel).

These results together show that *S. uberis*, either as live pathogen or as heat-killed particle avoids almost completely triggering any TLR-signaling. The particles are unrecognizable by TLR2 and this deficit is not efficiently compensated for by any other pathogen receptor.

### LTAs from *S. uberis* activated immune gene expression and NF-κB in pbMEC, but not through TLR2 activation

**Figure 4:**
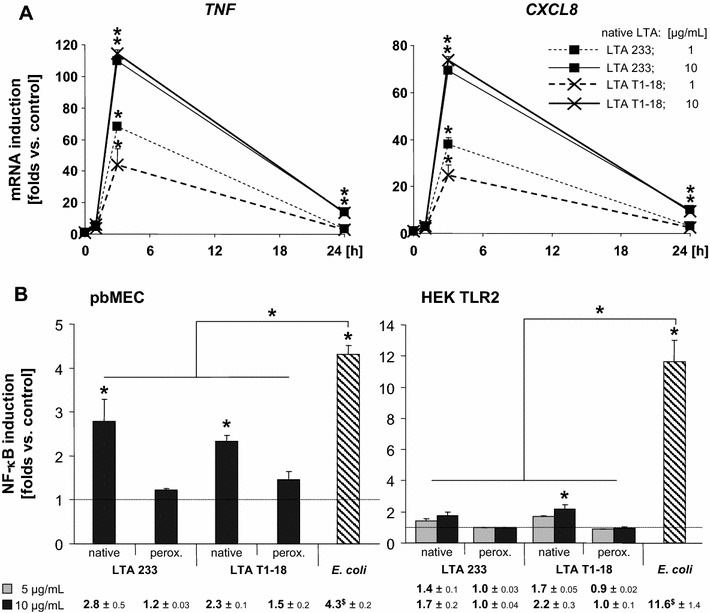
**LTA significantly activated immune gene expression and NF-κB in pbMEC, but not through TLR2 activation.** **A** pbMEC were stimulated with 10 µg/mL of the respective LTA preparation for the time, as indicated (abscissa). *TNF* and *CXCL8* mRNA concentrations were measured from duplicate assays, normalized against the *CLIC1* reference and expressed as multiples of the concentration from unstimulated controls (*p < 0.05). **B** The same LTA preparations or their peroxide treated derivatives were used to stimulate pbMEC cultures having previously been transfected with the ELAM driven NF-κB reporter gene (left hand panel, stimulated with 10 µg/mL LTA) or HEK293 cells, having been co-transfected with that NF-κB reporter and the construct expressing the bovine TLR2 receptor. The different dose of the challenge substance is indicated. ^$^ *E. coli* stimulation was with 30 µg/mL. These experiments are representative for three (left panel) or two (right panel) each assayed in triplicate.

Given the almost absent stimulatory capacity of the *S. uberis* particles, we wondered if LTAs as major cell envelope components would stimulate immune gene expression in pbMEC. LTAs were isolated from two different strains. Both preparations strongly activated expression of our candidate immune genes. The examples shown in Figure 4A and Table 2 also demonstrate that there was no difference between the LTAs isolated from the strains 233 and T1–18.

**Table 2:** **Extent and kinetics of modulated mRNA concentrations after stimulating pbMEC with native LTA from** ***S. uberis*** **strain 233 and T1–18.**

Gene	LTA from strain	Concentration (µg/mL)	Time		
			1 h	3 h	24 h
*TNF*	233	1	4.6 ± *0.3*	***68*** ± *1.8*	3.2 ± *0.2*
		10	5.7 ± *0.0*	***110*** ± *2.9*	**14.1** ± *0.2*
	T1–18	1	3.6 ± *0.3*	**44** ± *10.0*	2.8 ± *0.1*
		10	6.1 ± *0.3*	**114** ± *2.2*	**13.4** ± *0.3*
*IL6*	233	1	1.2 ± *0.0*	**10.2** ± *0.8*	1.9 ± *0.1*
		10	1.3 ± *0.1*	**18.5** ± *0.2*	**4.3** ± *0.04*
	T1–18	1	1.2 ± *0.1*	**7.1** ± *1.7*	1.7 ± *0.1*
		10	1.2 ± *0.1*	**18.5** ± *0.6*	**4.1** ± *0.1*
*CXCL8*	233	1	2.9 ± *0.05*	**38** ± *2.7*	3.2 ± *0.0*
		10	3.2 ± *0.1*	**69** ± *1.1*	**10.2** ± *0.2*
	T1–18	1	2.1 ± *0.1*	**25** ± *4.3*	2.5 ± *0.3*
		10	3.2 ± *0.2*	**74** ± *1.7*	**9.3** ± *0.1*
*CCL5*	233	1	0.9 ± *0.01*	**6.6** ± *0.3*	**6.0** ± *0.1*
		10	1.1 ± *0.1*	**14.7** ± *0.6*	**28** ± *1.1*
	T1–18	1	1.0 ± *0.01*	3.7 ± *1.2*	**4.9** ± *0.4*
		10	1.0 ± *0.1*	**13.4** ± *1.2*	**33** ± *2.8*
*NOS2A*	233	1	1.0 ± *0.04*	**55** ± *3.2*	2.0 ± *0.1*
		10	1.0 ± *0.02*	**119** ± *5.4*	7.3 ± *0.1*
	T1–18	1	0.9 ± *0.13*	**31** ± *12.8*	1.7 ± *0.1*
		10	1.0 ± *0.05*	**112** ± *9.7*	6.1 ± *0.3*
*LAP*	233	1	1.0 ± *0.08*	2.9 ± *0.08*	**17** ± *3.5*
		10	1.0 ± *0.04*	4.6 ± *0.48*	**47** ± *1.0*
	T1–18	1	0.9 ± *0.05*	2.2 ± *0.22*	**11** ± *1.4*
		10	1.0 ± *0.04*	4.2 ± *0.03*	**39** ± *0.7*
*SAA3*	233	1	1.1 ± *0.02*	34 ± *4.6*	31 ± *2.2*
		10	1.1 ± *0.00*	**50** ± *0.6*	**124** ± *30*
	T1–18	1	1.1 ± *0.02*	23 ± *6.9*	**45** ± *8.0*
		10	1.2 ± *0.01*	**50** ± *1.6*	**119** ± *18*
*CYP1A1*	233	1	1.0 ± *0.02*	1.7 ± *0.1*	1.0 ± *0.04*
		10	2.1 ± *0.03*	**25** ± *1.5*	1.5 ± *0.1*
	T1–18	1	1.0 ± *0.03*	1.4 ± *0.2*	1.3 ± *0.2*
		10	1.8 ± *0.11*	**20** ± *0.6*	1.8 ± *0.1*

pbMEC were stimulated with 1 or 10 µg/mL LTA for the indicated time. Values are means (±SEM) of fold change relative to unstimulated control from two biological replica experiments, each assayed in duplicate; bold numbers represent significant regulation

Strong induction of immune gene expression in pbMEC was accompanied by significant activation of NF-κB factors (Figure 4B, left hand panel). Inactivation of potentially co-isolated lipoprotein components through peroxide treatment significantly reduced the NF-κB stimulation by these LTA preparations. The slight residual NF-κB activation was statistically insignificant. We also investigated if other components of the *S. uberis* strain 233 cell envelope (lipoproteins, lipids, glycolipids) might activate NF-κB factors in pbMEC. None of those components significantly activated NF-κB in pbMEC (Additional file 5).

The NF-κB stimulatory activity of the LTA preparations was not mediated through TLR2. This became clear by analyzing the NF-κB stimulatory activity of these preparations in the HEK293 reconstitution system of TLR2 activation (Figure 4B, right hand panel). The HEK293 cells were co-transfected with the NF-κB driven luciferase reporter gene and a vector expressing the bovine TLR2 receptor. Stimulations with different dose of the native LTA preparations only slightly elevated the level of active NF-κB factors. Peroxide treatment of the same LTA preparations abolished completely their capacity to activate NF-κB. *E. coli*, on the other hand activated NF-κB in these experiments very strongly, by more than 11 fold.

These data together show that the cell envelope component LTA isolated from *S.uberis* is in principal recognizable by receptors of the pbMEC relevant for triggering an immune alert.

### *Streptococcus uberis* strongly activated immune gene expression and NF-κB factors in murine RAW264.7 macrophage model cells

**Figure 5:**
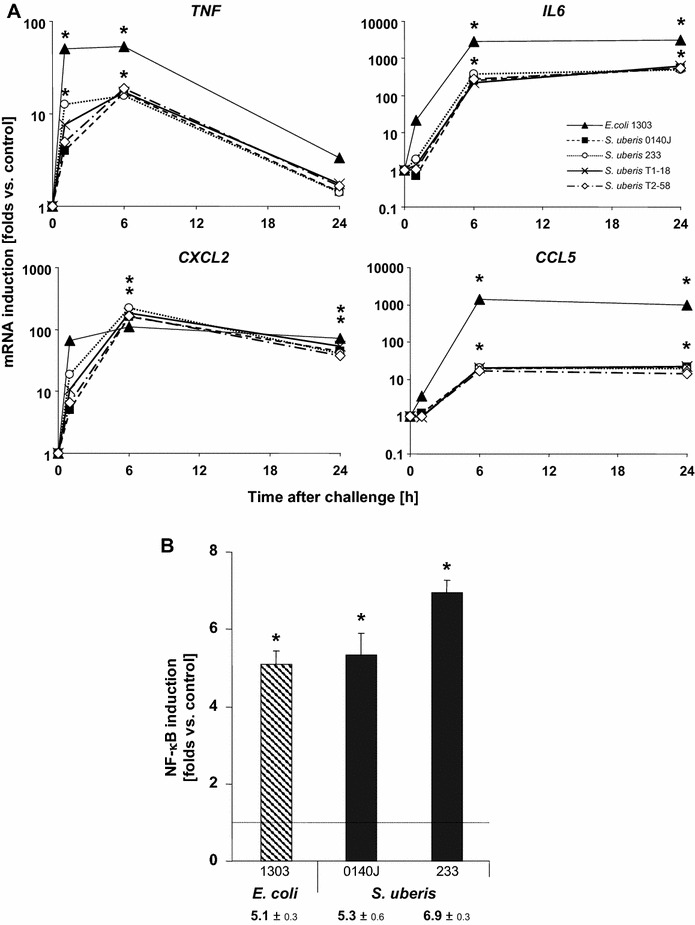
***Streptococcus uberis*** **particles significantly activated immune gene expression and NF-κB in macrophages.** **A** Murine macrophage RAW 264.7 cells were stimulated for the time as indicated (abscissa) with 30 µg/mL of heat-killed particles of *E. coli*_1303_ or of the *S. uberis* strains 0140J, 233, T1–18, and T2–58. *TNF*, *IL6*, *CXCL2*, and *CCL5* mRNA concentrations were measured from duplicate assays and expressed as multiples of the concentration from unstimulated controls (*p < 0.05). **B** RAW 264.7 cells were transfected with the ELAM driven reporter gene construct (100 ng) and stimulated with 30 µg/mL of heat-killed particles of *E. coli*_1303_ or of the *S. uberis* strains 0140J or 233 for 24 h. Renilla activity was measured from those cell lysates and expressed as multiple of the respective unstimulated control (ordinate, tabulated values below the graph, mean ± SEM, *n* = 2 independent experiments, each assayed in triplicate, * *p* < 0.05).

The poor immune stimulatory capacity of *S. uberis* was peculiar for pbMEC. This was found by stimulating the murine macrophage model cell line RAW264.7 with the same *S. uberis* preparations as used in the pbMEC stimulations. Challenging these cells with heat-killed particles from three different *S. uberis* strains resulted in a significant and strong induction of all immune genes examined (Figure 5A). They induced expression of the *CXCL2* encoding gene to a similar extent as *E. coli*. The other three genes analyzed (*TNF*, *IL6*, *CCL5*) were also all significantly induced by all three *S. uberis* strains, albeit to a lesser extent than by *E. coli*. These inductions of immune gene expression were paralleled by strong and significant activation of NF-κB factors in these cells, similarly as caused by challenging them with *E. coli* (Figure 5B).

## Discussion

It is still unclear whether or not the physiological outcome of an udder infection with *S. uberis* as either clinical or sub-clinical mastitis relates to the divergent genotypes of respective pathogen. The controversial literature in this regard was recently extensively reviewed by Zadoks et al. [2]. The overarching goal of our study was therefore to examine if the respective mastitis physiology might be caused by *S. uberis* strain specific differences in the interaction with the MEC. As a first step, we used here for most of the experiments preparations of heat killed *S. uberis* particles from diverse strains to ensure good technical reproducibility. It is known that PAMPs activate immune functions in MEC very quickly (<1 h; [48]). Our experimental setting thus mainly focused on eventual differences in the passive—PAMP-related—immune induction capacity of the various *S. uberis* strains and ignored the possibly very crucial effects of virulence factors secreted by the live pathogens. Their effect emerges at later times during host pathogen interaction, after their accumulation in high enough concentrations in the alveolar fluid to significantly modulate the MEC immune responsiveness.

We conducted the study on the background of our broad experiences regarding the pathogen-species specific immune response of the pbMEC model system towards challenges with *E. coli* and *S. aureus* mastitis pathogens under various experimental conditions [17, 48, 49, 54].

### It is a prevalent property of *S. uberis* to not trigger immune functions in MEC

Our key observation is that the MEC generally does not mount an adequate immune response against *S. uberis*. This general immune unresponsiveness of these cells occurred in our experiments, albeit that the cells had perceived indeed the xenobiotic stress caused by the presence of the bacteria in the environment as shown by the strong, uniform and dose dependent induction of *CYP1A1* expression by heat-killed bacteria, irrespective of their species. Using heat-killed preparations of 14 different strains we found (1) that none of these strains significantly induced any substantial immune gene expression and (2) no indication of any significant quantitative difference in the responses in pbMEC induced by isolates from clinical or subclinical cases of mastitis. Moreover, this uniform escape from alerting immune functions in this specific cell type of the host was independent from the capacity of *S. uberis* for capsule formation (0140J forms a capsule while 233 does not [14]; similar reaction observed against 0140J and its mutant 0140J_*Δhas*_) or the virulent vs. avirulent phenotype of the *S. uberis* pathogens.

The lack of MEC responsiveness against the challenging pathogens was not due to gross surface alterations associated with the heat inactivation of the pathogens, since short-term co-culture with live pathogens and their subsequent inactivation with gentamicin conceivably left the surface structure of the pathogens unchanged and nevertheless did not cause a much stronger reaction. Gram-positive bacteria are known to evade immune recognition through masking with serum components [55–57]. However, our control experiment challenging the MEC in serum free medium proved that no such mechanism was responsible for the general unresponsiveness in the current study. The slightly enforced reactivity of the MEC against the live pathogens might be due the presence of some small RNA molecules which are found on the surface of live pathogens but which might have been washed away during the heat inactivation procedure. Such “vita-PAMPs” were found to eventually induce stronger expression of some immune genes (examples *IFN*-*β*, *IL1*-*β*) but not of others, such as *IL6* [58]. Indeed, small RNA molecules have also been found adhering to the surface of *S. uberis* (JA Leigh, personal communication).

Considering all these controls we can therefore exclude technical errors as cause for the observed immune unresponsiveness of the MEC against a wide variety of *S. uberis* strains which all had been isolated from the bovine mammary gland. However, our data are at variance with a few reports that some particular strains did indeed induce some immune reaction in pbMEC cultures [14, 29]. Such strains must be rare since we did not find a single isolate with these properties in our collection of more than twenty different strains.

### *Streptococcusuberis* does not activate substantial TLR-signaling in MEC

We identify complete absence of any *S. uberis* induced TLR-signaling in MEC as a major molecular cause for the failure of these cells to mounting an immune defense against pathogens of this species. This was revealed since neither challenging the pbMEC with heat-killed nor with live pathogens increased the level of active NF-κB factors. Activation of this transcription factor complex however is the integrating indicator for PRR-signaling, including TLR- and NOD- signaling [30, 31]. We also validated in particular that *S. uberis* does not activate TLR2-signaling confirming previous reports [41]. Moreover, our results demonstrate that the failure to activate PRR signaling is peculiar to *S. uberis* from among the streptococcal species complex. *S. agalactiae* and *S. dysgalactiae* activated the bovine TLR2 receptor in HEK293 cells. TLR2 activation through *S. agalactiae* was also previously reported [37], but has not been found by two other groups [40, 41]. No explanation can be given to resolve this discrepancy.

### Purified LTA from *S. uberis*, but not other cell envelope components can in principle activate immune functions in MEC

We examined the immune stimulatory properties of LTA, glycolipids, lipids and lipoproteins isolated from *S. uberis*. Only LTA was found to significantly stimulate an immune response in MEC. LTA is an integral component of the cell envelope of Gram-positive bacteria [59]. It is long known as having immune stimulatory properties, but it still is questionable which PRR is its cognate receptor. The controversial debate regarding the role of TLR2 for LTA recognition has recently been summarized [60]. Our observation that isolated LTA from two different *S. uberis* strains strongly induced immune gene expression in MEC to similar extent is well in line with a wealth of reports showing the immune stimulatory function of such molecules [47, 61, 62]. Nuclear magnetic resonance spectroscopy experiments showed no structural differences between the LTAs isolated from strain 233 and T1-18 [43]. Detailed structural analysis of LTA from strain 233 revealed that this LTA may be typical for streptococcal LTA, since its structure was found to be very similar to LTAs prepared from *S. agalactiae* and *S. dysgalactiae* [46]. Significant NF-κB activation through the *S. uberis* derived LTA preparations in MEC strongly suggests activation of PRR-mediated signaling, but not involving TLR2 according to our data. Activation of NF-κB was associated with the integrity of the native LTA preparation. These are known to be possibly contaminated with co-purifying lipoproteins [60]. The immune stimulatory properties of LTA and lipoproteins can be inactivated by H_2_O_2_ oxidation [47]_,_ and this treatment abolished any significant NF-κB activation in MEC through our LTA preparations.

Clearly, our data do not allow identification of the LTA receptor. Obviously, TLR2 is not involved in mediating the LTA elicited response. This is in line with previous reports that chemically synthesized LTA does not activate TLR2 signaling [63]. Nevertheless, our data show that isolated cell envelope components of *S. uberis* may indeed strongly activate immune functions in MEC. This suggests, in turn that such immune stimulatory components are structurally arranged in the cell envelope such that they are unrecognizable to the relevant PRRs of the MEC. Hence, the design of the outer surface of the *S. uberis* cell apparently provides a “magic hood” preventing recognition of the pathogen by the MEC.

### *S. uberis* activates immune response in macrophages

We were curious to learn if the sluggish immune response towards *S. uberis* was peculiar to the MEC phenotype and therefore stimulated the murine macrophage model cell RAW264.7 with the very same *S. uberis* preparations as used before. We validated in a separate study (Günther J, Koy, M, Schuberth, HJ, Seyfert HM; unpublished, manuscript in preparation) that the pathogen-species specific immune response of the RAW264.7 is very similar as recorded from primary bovine monocyte derived macrophages (MDM). There is some evidence that *S. uberis* may be phagocytosed by macrophages [64, 65], but to the best of our knowledge quantification of the immune response of macrophages against *S. uberis* has not yet been reported. Using preparations from four different strains we found invariantly that they induced a strong expression of cytokine and chemokine encoding genes. This was very likely caused by strong PRR mediated signaling, since the challenges strongly activated the NF-κB factor complex. These data show very clearly that professional immune cells are capable of recognizing *S. uberis* as a threatening pathogen. One of the key functions of these cells is to engulf and digest pathogens. Phagocytosis is not a key function of epithelial cells, such as MEC. Hence, it was perhaps not surprising to find that eventually invaded *S. uberis* pathogens may persist as structurally intact particles inside of MEC [66]. However, intra-cellular digestion of Gram-positive bacteria and subsequent activation of intracellular PRRs, such as TLR9 or NOD2 was found in many cases to be pivotal for mounting an adequate immune response against those invaders (see review [55]). Hence, data from this study here highlight in turn that the failure of the MEC to respond to the *S. uberis* challenge is specifically related to the MEC phenotype.

In summary, we demonstrated here that all the strains from our large collection of *S. uberis* isolates from clinical and subclinical cases of mastitis evaded the immune surveillance of the MEC, representing by far the most abundant first line sentinels of the udder. Failure to activating their immune alert early on after infection explains the commonly observed belated and weak onset of udder inflammation during *S. uberis* mastitis. We proved, on the other hand that macrophages can indeed mount a vigorous immune response against *S. uberis*. Hence, our data collectively imply that the observed large fraction of subclinical mastitis associated with *S. uberis* infections is determined by the pathogen-species specific immune response of MEC. The sometimes occurring severe cases of clinical mastitis after *S. uberis* infection may relate to specific properties of the individual cow, conceivably including an altered setting and equipment of the udder with resident professional immune cells.

## Additional files

10.1186/s13567-015-0287-8 **Sequences of the oligonucleotide primers used for real-time PCR quantification.** Primer sequences and source files for the respective genes are indicated.
10.1186/s13567-015-0287-8 **Extent and kinetics of modulated mRNA concentrations after stimulating pbMEC with heat-killed** ***E. coli***_**1303**_ **or four different** ***S. uberis*** **strains for 6 and 24 h.** Values are means of fold changes of the respective mRNA concentration (relative to the unstimulated control culture) from two biological replica experiments (± SEM), each assayed in duplicate; bold numbers represent significant regulation.
10.1186/s13567-015-0287-8 **Extent and kinetics of modulated mRNA concentrations after stimulating pbMEC in serum free medium with** ***E. coli***_**1303**_, ***S. aureus*** **strain 1027 or** ***S. uberis*** **strain 233.** Values are means from two biological replica experiments (± SEM) of fold changes relative to unstimulated control; bold numbers represent significant regulation (Anova, Bonferroni post-tests).
10.1186/s13567-015-0287-8 ***S. uberis*** **pretreatment of pbMEC did not change the immune response against a subsequent E. coli challenge**. pbMEC were pretreated (primed) with 30 µg/mL heat-killed particles from *S. uberis* strain 0140J or 233 for one hour. Subsequently the cells were washed three times with PBS and cultivated in normal growth medium (0140J priming, 233 priming) or were challenged with 3 µg/mL heat-killed particles from *E. coli* strain 1303 (0140J priming + *E. coli*, 233 priming + *E. coli*) for another 1 h, 3 h or 24 h. To analyze the response against *E. coli* without priming pbMEC were cultivated one hour in normal growth medium, washed three times with PBS and were challenged with 3 µg/mL *E. coli* particles only for 1 h, 3 h or 24 h (*E. coli*). Cells were harvested at the end of the experiment and total RNA was prepared. *TNF*, *CXCL8*, *NOS2A*, and *LAP* mRNA concentrations were measured with RT-qPCR and expressed as multiples of the concentration from unstimulated controls. Data are from a single experiment, assayed in duplicate.
10.1186/s13567-015-0287-8 **Other membrane anchored components of the cell envelope from** ***S. uberis*** **strain 233 did not significantly activate NF-κB in pbMEC**. pbMEC were transfected with the ELAM driven reporter gene construct (100 ng) and stimulated with 10 µg/mL of the indicated *S.* *uberis* component or 30 µg/mL *E. coli*_1303_ for 24 h. The luciferase activity was measured from cell lysates and normalized against their protein concentration. Values are expressed as fold increase above the level of the unstimulated control (ordinate). Each transfection was run in duplicate and assayed from triplicate challenges. (**p* < 0.05). Components of the *S. uberis* cell envelope were prepared by bead disruption of the cells as described for the LTA preparation in the Material and Methods section of the main text. Lipoproteins were obtained by Triton X-114 phase partitioning of the membrane fraction as described [67]. Lipids were extracted according to the method of Bligh and Dyer [68]. Thin Layer Chromatography (TLC) was used to identify glycolipid in the total lipid extract. Samples were developed using a mixture of chloroform/methanol/H_2_O (65/25/4, v/v/v) and visualized with Hanessian’s and α-naphtol stain. Three glycolipids G1, G2 and G3 were identified. To isolate these glycolipids the crude lipid extract was fractionated on activated Silica Gel 60 and glycolipids were successively eluted with chloroform/methanol in the ratios of 9.5:0.5 (G1), 9:1 (G2), and 1:1 (G3). Those fractions were dried and further purified by preparative TLC to obtain pure specific substances. In the NF-κB assay lipoproteins were used untreated (native), proteinase K (PK) or H_2_O_2_ treated (perox). Furthermore NF-κB activation capacity of water and inter phase from the lipid extraction procedure and of the three glycolipids was examined and compared to a challenge with *E. coli*. The data regarding the lipoproteins show that the slight NF-κB activation is not specifically related to lipoproteins, since both, proteinase K as well as H_2_O_2_ treatment destroys the structural integrity of such molecules.

## Abbreviations

LTA: lipoteichoic acid
NF-κB: nuclear factor kappa-light-chain-enhancer of activated B-cells
PRR: pattern recognition receptor
TLR: toll-like receptor
RT-qPCR: reverse transcription quantitative PCR
