# Supplementary material for: Streptococcus uberis strains isolated from the bovine mammary gland evade immune recognition by mammary epithelial cells, but not of macrophages
Source: Vet Res. 2016 Jan 7;47:13. doi: 10.1186/s13567-015-0287-8 (PMC4704416; doi:10.1186/s13567-015-0287-8)
Supplement: Supplementary file 1 — 10.1186/s13567-015-0287-8 Sequences of the oligonucleotide primers used for real-time PCR quantification. Primer sequences and source files for the respective genes are indicated. [file 13567_2015_287_MOESM1_ESM.docx]

**Additional file 3** **Sequences of the oligonucleotide primers used for real-time PCR quantification.**

| **Species** | **Gene** | **GenBank no.** | **Primer sequence (5´–3´)** | **Amplificate size (bp)** |
| --- | --- | --- | --- | --- |
| ***Bos taurus*** | |  |  |  |
|  | *TNF* | NM_173966.2 | CTTCTGCCTGCTGCACTTCG * | 156 |
|  |  |  | GAGTTGATGTCGGCTACAACG * |  |
|  |  |  |  |  |
|  | *IL1A* | NM_174092 | GGCCAAAGTCCCTGACCTCT | 224 |
|  |  |  | CTGCCACCATCACCACATTC |  |
|  |  |  |  |  |
|  | *IL1B* | NM_174093.1 | AACCGAGAAGTGGTGTTCTGC | 167 |
|  |  |  | TTGGGGTAGACTTTGGGGTCT |  |
|  |  |  |  |  |
|  | *IL6* | NM_000600.3 | GGAGGAAAAGGACGGATGCT | 227 |
|  |  |  | GGTCAGTGTTTGTGGCTGGA |  |
|  |  |  |  |  |
|  | *CXCL8* | NM_173925 | CCTCTTGTTCAATATGACTTCCA | 170 |
|  |  |  | GGCCCACTCTCAATAACTCTC |  |
|  |  |  |  |  |
|  | *CCL5* | NM_175827 | TCCCCATATGCCTCGGAC | 229 |
|  |  |  | TCGCACCCACTTCTTCTCTG |  |
|  |  |  |  |  |
|  | *CCL20* | NM_174263.2 | CAGCAAGTCAGAAGCAAGCAA | 179 |
|  |  |  | CCCACTTCTTCTTTGGATCTGC |  |
|  |  |  |  |  |
|  | *NOS2A* | NM_001076799 | ACAGGATGACCCCAAACGTC | 188 |
|  |  |  | TCTGGTGAAGCGTGTCTTGG |  |
|  |  |  |  |  |
|  | *SAA3* | NM_181016.3 | CTTTCCACGGGCATCATTTT | 188 |
|  |  |  | CTTCGGGCAGCGTCATAGTT |  |
|  |  |  |  |  |
|  | *LAP* | NM_203435 | AGGCTCCATCACCTGCTCCTT | 182 |
|  |  |  | CCTGCAGCATTTTACTTGGGCT |  |
|  |  |  |  |  |
|  | *CYP1A1* | XM_002696635  XM_005222018 | TCCGTTACCTGCCCAACACT | 170 |
|  |  |  | CTCGTCCAGCCTCTTGTCCT |  |
|  |  |  |  |  |
|  | *CLIC* | NM_001015608 | GTCTCAGTCCGCCTCTTGGT | 153 |
|  |  |  | AGAACAACCGCAGGTCGAAT |  |
| ***Mus musculus*** | |  |  |  |
|  | *TNF* | NM_013693.3  NM_001278601.1 | ccgtcagccgatttgctatc | 160 |
|  |  |  | agttggtcccccttctccag |  |
|  |  |  |  |  |
|  | *IL6* | NM_031168.1 | AACGATGATGCACTTGCAGA | 195 |
|  |  |  | CTGAAGGACTCTGGCTTTGTC |  |
|  |  |  |  |  |
|  | *CXCL2* | NM_009140.2 | actctcaagggcggtcaaaa | 191 |
|  |  |  | caggtacgatccaggcttcc |  |
|  |  |  |  |  |
|  | *CCL5* | NM_013653.3 | CCCTCACCATCATCCTCACT | 181 |
|  |  |  | CGAGTGACAAACACGACTGC |  |
|  |  |  |  |  |
|  |  |  |  |  |
| *upper line: forward-, lower line, reverse-primer; source files are indicated. | | | |  |
