# Supplementary material for: Streptococcus uberis strains isolated from the bovine mammary gland evade immune recognition by mammary epithelial cells, but not of macrophages
Source: Vet Res. 2016 Jan 7;47:13. doi: 10.1186/s13567-015-0287-8 (PMC4704416; doi:10.1186/s13567-015-0287-8)
Supplement: Supplementary file 2 — 10.1186/s13567-015-0287-8 Extent and kinetics of modulated mRNA concentrations after stimulating pbMEC with heat-killed E. coli 1303 or four different S. uberis strains for 6 and 24 h. Values are means of fold changes of the respective mRNA concentration (relative to the unstimulated control culture) from two biological replica experiments (± SEM), each assayed in duplicate; bold numbers represent significant regulation. [file 13567_2015_287_MOESM2_ESM.docx]

**Additional file 4** **Extent and kinetics of modulated mRNA concentrations after stimulating pbMEC with heat-killed *E. coli* strain 1303 or four different *S. uberis* strains for 6 and 24 h.**

|  |  |  |  | | | |  |  |  |
| --- | --- | --- | --- | --- | --- | --- | --- | --- | --- |
| **Gen** | **Time** | ***E. coli*** | ***S. uberis*** | | | | |  |  |
|  | **[h]** | **1303** | **O140J** | **T1-18** | **T2-58** | **233** | |  |  |
|  |  |  |  |  |  |  | |  |  |
| ***TNF*** | 6 | **226 ±** 111 | 1.6 ± *0.2* | 1.6 ± *0.1* | 1.4 ± *0.3* | 1.4 ± *0.0* | |  |  |
|  | 24 | **100** ± *87* | 1.3 ± *0.2* | 1.6 ± *0.2* | 1.1 ± *0.2* | 1.0 ± *0.0* | |  |  |
|  |  |  |  |  |  |  | |  |  |
| ***IL6*** | 6 | **22** ± *10* | 1.1 ± *0.0* | 1.1 ± *0.2* | 0.9 ± *0.1* | 1.0 ± *0.2* | |  |  |
|  | 24 | **22** ± *6* | 1.1 ± *0.1* | 1.3 ± *0.4* | 1.3 ± *0.5* | 1.0 ± *0.0* | |  |  |
|  |  |  |  |  |  |  | |  |  |
| ***CXCL8*** | 6 | **141** ± *19* | 1.2 ± *0.1* | 1.1 ± *0.1* | 1.1 ± *0.1* | 1.2 ± *0.1* | |  |  |
|  | 24 | **71** ± *41* | 1.0 ± *0.1* | 1.0 ± *0.1* | 1.0 ± *0.0* | 1.0 ± *0.1* | |  |  |
|  |  |  |  |  |  |  | |  |  |
| ***CCL5*** | 6 | 171 ± *75* | 0.9 ± *0.3* | 0.7 ± *0.1* | 0.7 ± *0.1* | 0.8 ± *0.1* | |  |  |
|  | 24 | 259 ± *114* | 0.9 ± *0.1* | 0.8 ± *0.1* | 0.7 ± *0.1* | 0.9 ± *0.0* | |  |  |
|  |  |  |  |  |  |  | |  |  |
| ***NOS2A*** | 6 | **274** ± *245* | 0.8 ± *0.1* | 0.9 ± *0.2* | 0.7 ± *0.0* | 0.8 ± *0.2* | |  |  |
|  | 24 | **52** ± *30* | 1.1 ± *0.2* | 1.1 ± *0.3* | 1.0 ± *0.1* | 1.0 ± *0.2* | |  |  |
|  |  |  |  |  |  |  | |  |  |
| ***LAP*** | 6 | 12 ± *11* | 1.0 ± *0.0* | 1.2 ± *0.3* | 0.9 ± *0.1* | 1.0 ± *0.2* | |  |  |
|  | 24 | 130 ± *55* | 1.1 ± *0.1* | 1.3 ± *0.3* | 1.0 ± *0.2* | 0.7 ± *0.1* | |  |  |
|  |  |  |  |  |  |  | |  |  |
| ***SAA3*** | 6 | 210 ± *109* | 0.9 ± *0.2* | 1.0 ± *0.4* | 0.8 ± *0.2* | 1.0 ± *0.3* | |  |  |
|  | 24 | 482 ± *506* | 1.3 ± *0.6* | 1.7 ± *1.1* | 1.0 ± *0.4* | 1.2 ± *0.7* | |  |  |
|  |  |  |  |  |  |  | |  |  |
| ***CYP1A1*** | 6 | **64** ± *48* | **72** ± *44* | **74** ± *44* | **57** ± *37* | **83** ± *46* | |  |  |
|  | 24 | 1.3 ± *0.1* | 1.1 ± *0.4* | 1.2 ± *0.5* | 0.9 ± *0.2* | 1.3 ± *0.7* | |  |  |
|  |  |  |  |  |  |  | |  |  |

Values are means of fold changes of the respective mRNA concentration (relative to the unstimulated control culture) from two biological replica experiments (± SEM), each assayed in duplicate; bold numbers represent significant regulation.
