# Supplementary material for: Streptococcus uberis strains isolated from the bovine mammary gland evade immune recognition by mammary epithelial cells, but not of macrophages
Source: Vet Res. 2016 Jan 7;47:13. doi: 10.1186/s13567-015-0287-8 (PMC4704416; doi:10.1186/s13567-015-0287-8)
Supplement: Supplementary file 3 — 10.1186/s13567-015-0287-8 Extent and kinetics of modulated mRNA concentrations after stimulating pbMEC in serum free medium with E. coli 1303, S. aureus strain 1027 or S. uberis strain 233. Values are means from two biological replica experiments (± SEM) of fold changes relative to unstimulated control; bold numbers represent significant regulation (Anova, Bonferroni post-tests). [file 13567_2015_287_MOESM3_ESM.docx]

**Additional file 5** **Extent and kinetics of modulated mRNA concentrations after stimulating pbMEC in serum free medium with *E. coli* strain 1303, *S. aureus* strain 1027 or *S. uberis* strain 233.**

|  |  |  |  |  |
| --- | --- | --- | --- | --- |
| **Gene** | **Time** |  | **Pathogen** |  |
|  |  |  |  |  |
|  |  | ***E. coli***  **strain 1303** | ***S. aureus* strain 1027** | ***S. uberis***  **strain 233** |
|  |  |  |  |  |
| ***TNF*** | **1 h** | 14 ± 3.4 | 4.2 ± 0.6 | 0.9 ± 0.0 |
|  | **3 h** | **152** ± 15 | **57** ± 6.6 | 1.8 ± 0.2 |
|  | **24 h** | **84** ± 2.2 | **11** ± 0.7 | 2.3 ± 0.4 |
|  |  |  |  |  |
| ***IL1A*** | **1 h** | 4.6 ± 1.1 | 2.4 ± 0.3 | 2.1 ± 0.2 |
|  | **3 h** | **154** ± 17 | **43** ± 3.9 | 2.6 ± 0.1 |
|  | **24 h** | **25** ± 0.3 | 3.3 ± 0.1 | 1.6 ± 0.0 |
|  |  |  |  |  |
| ***IL1B*** | **1 h** | 2.3 ± 1.1 | 1.7 ± 0.8 | 1.7 ± 0.6 |
|  | **3 h** | **335** ± 74 | **83** ± 18 | 1.1 ± 0.2 |
|  | **24 h** | **290** ± 45 | 7.3 ± 0.2 | 1.4 ± 0.2 |
|  |  |  |  |  |
| ***IL6*** | **1 h** | 1.5 ± 0.3 | 0.8 ± 0.0 | 0.9 ± 0.0 |
|  | **3 h** | **98** ± 19 | 18 ± 2.3 | 1.3 ± 0.1 |
|  | **24 h** | **25** ± 0.5 | 3.3 ± 0.0 | 1.6 ± 0.1 |
|  |  |  |  |  |
| ***CXCL8*** | **1 h** | 5.1 ± 0.9 | 1.1 ± 0.3 | 1.2 ± 0.2 |
|  | **3 h** | **238** ± 25 | **69** ± 3.8 | 2.7 ± 0.1 |
|  | **24 h** | **46** ± 7.5 | 5.3 ± 0.5 | 1.6 ± 0.2 |
|  |  |  |  |  |
| ***CCL20*** | **1 h** | 7.8 ± 1.8 | 1.2 ± 0.1 | 0.8 ± 0.1 |
|  | **3 h** | **1675** ± 33 | **393** ± 2.6 | 2.2 ± 0.1 |
|  | **24 h** | **101** ± 10 | 8.0 ± 1.2 | 1.6 ± 0.2 |
|  |  |  |  |  |
| ***CCL5*** | **1 h** | 1.1 ± 0.1 | 1.0 ± 0.1 | 0.9 ± 0.0 |
|  | **3 h** | 7.1 ± 0.8 | 1.3 ± 0.0 | 0.9 ± 0.0 |
|  | **24 h** | **250** ± 4.8 | **28** ± 1.4 | 1.7 ± 0.1 |
|  |  |  |  |  |
| ***NOS2A*** | **1 h** | 1.2 ± 0.2 | 1.1 ± 0.1 | 0.8 ± 0.1 |
|  | **3 h** | **461** ± 1.3 | **115** ± 1.5 | 1.0 ± 0.0 |
|  | **24 h** | **116** ± 8.5 | 14 ± 2.6 | 1.7 ± 0.3 |
|  |  |  |  |  |
| ***LAP*** | **1 h** | 1.4 ± 0.1 | 0.7 ± 0.2 | 1.0 ± 0.1 |
|  | **3 h** | **2.9** ± 0.9 | 1.6 ± 0.3 | 0.9 ± 0.2 |
|  | **24 h** | **25** ± 1.0 | **3.5** ± 0.1 | 1.0 ± 0.1 |
|  |  |  |  |  |
| ***CYP1A1*** | **1 h** | 81 ± 14 | 88 ± 11 | **151** ± 15 |
|  | **3 h** | **1062** ± 159 | **1074** ± 140 | **1017** ± 120 |
|  | **24 h** | 2.8 ± 0.2 | 3.6 ± 0.8 | 2.8 ± 0.4 |
|  |  |  |  |  |

Values are means from two biological replica experiments (± SEM) of fold changes relative to unstimulated control; bold numbers represent significant regulation (Anova, Bonferroni post-tests).
