# Supplementary figures and images for: Streptococcus uberis strains isolated from the bovine mammary gland evade immune recognition by mammary epithelial cells, but not of macrophages
Source: Vet Res. 2016 Jan 7;47:13. doi: 10.1186/s13567-015-0287-8 (PMC4704416; doi:10.1186/s13567-015-0287-8)

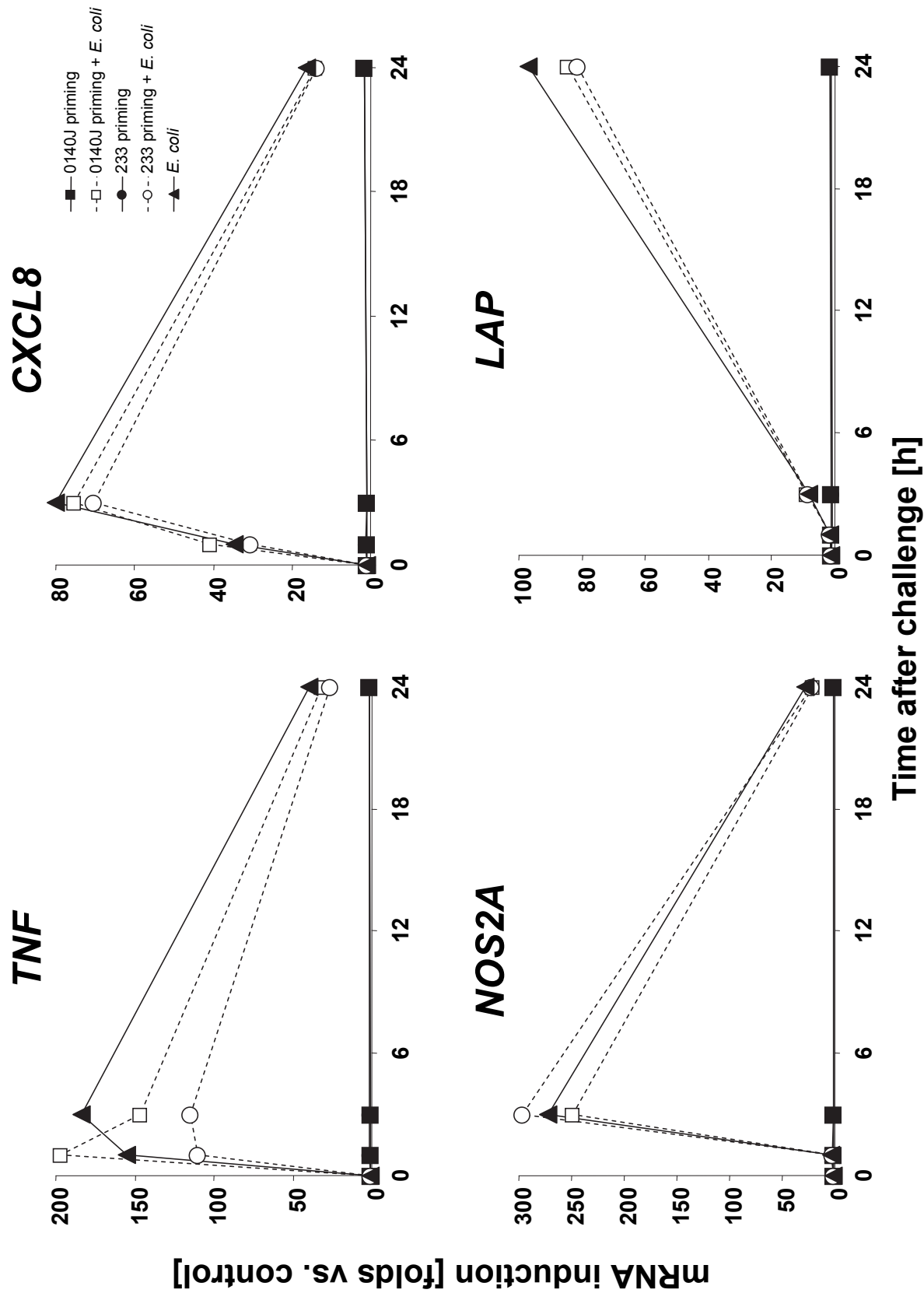

Supplement: Supplementary file 4 — 10.1186/s13567-015-0287-8 S. uberis pretreatment of pbMEC did not change the immune response against a subsequent E. coli challenge. pbMEC were pretreated (primed) with 30 µg/mL heat-killed particles from S. uberis strain 0140J or 233 for one hour. Subsequently the cells were washed three times with PBS and cultivated in normal growth medium (0140J priming, 233 priming) or were challenged with 3 µg/mL heat-killed particles from E. coli strain 1303 (0140J priming + E. coli, 233 priming + E. coli) for another 1 h, 3 h or 24 h. To analyze the response against E. coli without priming pbMEC were cultivated one hour in normal growth medium, washed three times with PBS and were challenged with 3 µg/mL E. coli particles only for 1 h, 3 h or 24 h (E. coli). Cells were harvested at the end of the experiment and total RNA was prepared. TNF, CXCL8, NOS2A, and LAP mRNA concentrations were measured with RT-qPCR and expressed as multiples of the concentration from unstimulated controls. Data are from a single experiment, assayed in duplicate. [file 13567_2015_287_MOESM4_ESM.pdf]

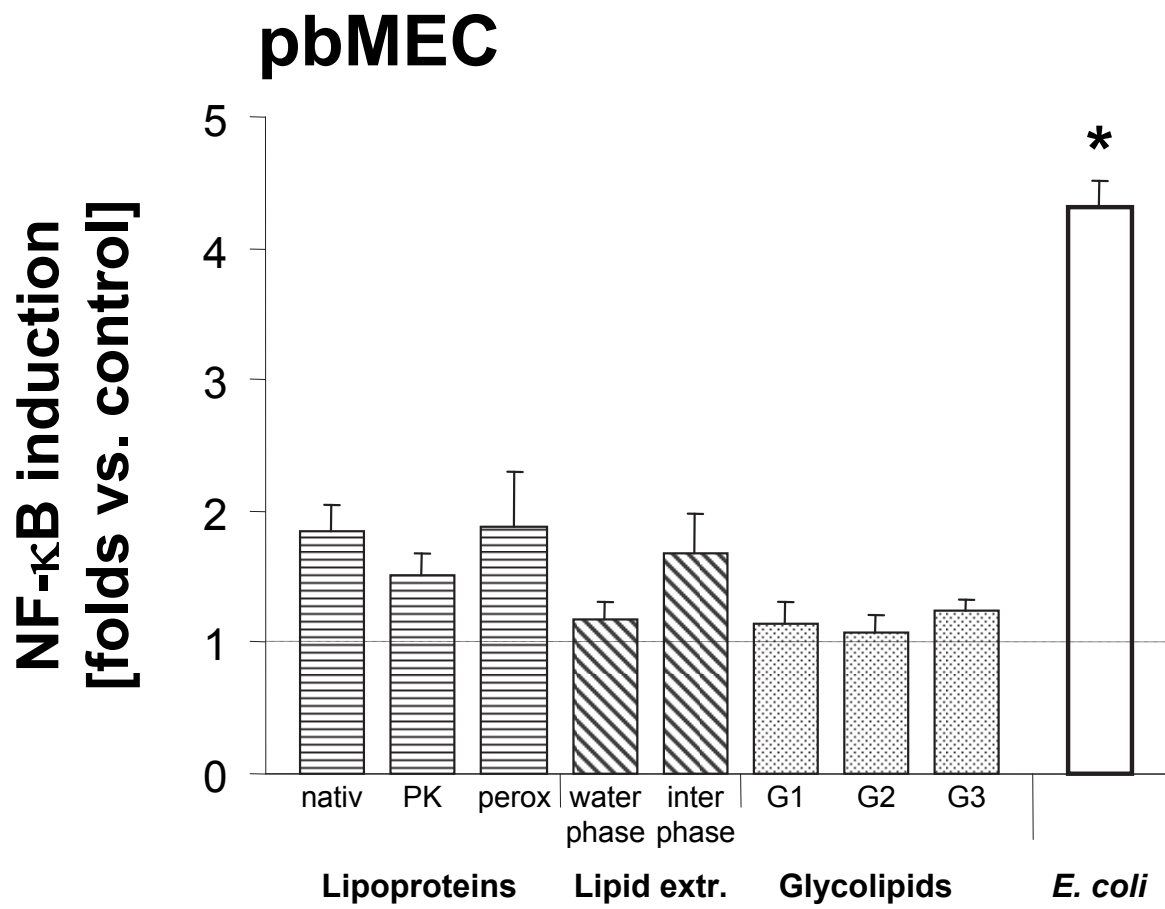

Supplementary figure 2

Supplement: Supplementary file 5 — 10.1186/s13567-015-0287-8 Other membrane anchored components of the cell envelope from S. uberis strain 233 did not significantly activate NF-κB in pbMEC. pbMEC were transfected with the ELAM driven reporter gene construct (100 ng) and stimulated with 10 µg/mL of the indicated S. uberis component or 30 µg/mL E. coli 1303 for 24 h. The luciferase activity was measured from cell lysates and normalized against their protein concentration. Values are expressed as fold increase above the level of the unstimulated control (ordinate). Each transfection was run in duplicate and assayed from triplicate challenges. (*p < 0.05). Components of the S. uberis cell envelope were prepared by bead disruption of the cells as described for the LTA preparation in the Material and Methods section of the main text. Lipoproteins were obtained by Triton X-114 phase partitioning of the membrane fraction as described [67]. Lipids were extracted according to the method of Bligh and Dyer [68]. Thin Layer Chromatography (TLC) was used to identify glycolipid in the total lipid extract. Samples were developed using a mixture of chloroform/methanol/H2O (65/25/4, v/v/v) and visualized with Hanessian’s and α-naphtol stain. Three glycolipids G1, G2 and G3 were identified. To isolate these glycolipids the crude lipid extract was fractionated on activated Silica Gel 60 and glycolipids were successively eluted with chloroform/methanol in the ratios of 9.5:0.5 (G1), 9:1 (G2), and 1:1 (G3). Those fractions were dried and further purified by preparative TLC to obtain pure specific substances. In the NF-κB assay lipoproteins were used untreated (native), proteinase K (PK) or H2O2 treated (perox). Furthermore NF-κB activation capacity of water and inter phase from the lipid extraction procedure and of the three glycolipids was examined and compared to a challenge with E. coli. The data regarding the lipoproteins show that the slight NF-κB activation is not specifically related to lipoproteins, since both, pr [file 13567_2015_287_MOESM5_ESM.pdf]
